# Thoughts on Chloroform

**Published:** 1852-11

**Authors:** 


*Thoughts on Chloroform.* By C. R. GILMAN, M. D., Professor of Obstetrics, &c., College of Physicians and Surgeons, New York.

If any apology is needed for him who attempts to interest the profession on the hackneyed theme of chloroform, it must be found in my case, in the deep feeling of responsibility which attaches to a public teacher, who is compelled by the results of his own experience to commend, and that earnestly, to his pupils, and thus, according to the measure of his influence, to extend the use of this agent, and yet can hardly open a medical periodical without learning that another and another life has been sacrificed to such use.

Is it to be expected—is it to be desired that such an one should turn from these harrowing narratives, and, satisfied that he has had no direct personal share in such horrors, dismiss the subject from his thoughts? Is not such an one to be pardoned if over zeal induce him to be "instant in season and out of season" in his efforts to prevent such deeds of death? Yet, how prevent them? Shall we banish anæsthetics from our materia medica—proscribe their use? Plainly this is impossible.—We cannot and *will not* give up the use of an agent which in our hands relieves suffering, cures disease, saves life—as we know chloroform does—because other men abuse it. Nobody expects us to do this with the forceps; and yet how often is health, and even life sacrificed by the careless abuse of the forceps! We must continue, then, to use, and those of us who are public teachers must continue to commend them. This, in the lecture room, is easy. There is no difficulty in *there* teaching the use and guarding against the abuse of anæsthetics: the rules are well settled. But who can assure us that, be we ever so careful, be our commendations ever so guarded, our influence may not be for evil in this matter? Even among our hearers, some may remember the commendation, and forget the cautions; and if the story chance to pass from mouth to mouth, what chance is there that more will be repeated than that Doctor this or Professor that recommended chloroform? Such are the reflections that have induced me to ask a place in your journal for a few thoughts on chloroform.

I shall say nothing in detail in favor of the practice, and therefore, perhaps, ought to premise that my confidence in its powers is undiminished, the number of cases in which I use it enlarges every day. Thus much for the bright, but my present business is with the dark side of the picture. First, then, let me state very briefly a case, which, as I suppose, throws some light on the varying degree in which it operates on different individuals. Mrs. J. was taken in labor early in the morning, January 17, 1852,—the child was born in a few hours, but almost immediately afterward the patient had severe convulsions. She was freely bled, had an enema, cold to the head, &c. I saw her in consultation, about three hours after the first fit; the convulsions were slight, patient very restless, consciousness *nul.* I thought it a case for chloroform, and the drug was sent for. During the absence of the messenger, a more careful examination of the pulse convinced me that she would bear more bleeding—the pulse, as I had anticipated, rose, and more than two pints were taken. Just as we were bandaging the arm, the chloroform was received, and at once administered. I gave it cautiously, my finger on the pulse, with no attempt to overwhelm the sensibility—a thing I never do; yet when the patient had taken four deep inspirations, the pulse fluttered—staggered—stopped. A dash of cold water, a few puffs of the face, and she gasped, her pulse staggered on—became regular, and all was well. Here the frightful symptoms were doubtless owing to the rapid absorption of the chloroform, consequent upon the profuse bleeding—the empty vessels were "*all agape,*" and every particle of chloroform was eagerly caught up. This influence of blood-letting in absorption is well known, yet I do not remember to have seen it alluded to in this connection. Its obvious importance will strike every one. It should lead the obstetrician to double caution in the use of anæsthetics where flooding has occurred, and the surgeon, in all bloody operations; especially if these latter are protracted, absorption will be rapid and danger proportionably great.

Thus much for a particular point: now let me offer a few suggestions on the great end and aim of this paper: How to diminish the danger. An d in the first place, let me urge on all who have not practical familiarity with the course and symptoms of anæsthesia, to use ether—sulphuric ether, I mean, for I verily believe that chloric ether is worse than chloroform; it is more likely to be used freely, i. e., carelessly; and thus used, *it will kill.* Use, then, sulphuric ether till you are at home in anæsthetics, yet, even then, beware lest this advantage lead you to carelessness with chloroform—*carelessness is death.* But it is not beginners only, who should prefer ether. The state of the patient may render one or the other preferable.

Without going into detail, I should say that exhaustion and prostration call for ether—great nervous excitement and vascular activity, chloroform.

Now, as to the extent to which the drug should be carried. Here I find myself at issue with the great mass of the surgeons. They all—all at least of whose doings I am cognizant, carry anæsthesia to the complete abolition of sensation. Is not this attempting too much? Never in natural labor, and very rarely in obstetric operations, do I go beyond a decided benumbing of sensibility. This may or may not be attended with loss of consciousness, for there is certainly no regular order of succession, or at least no invariable order observed. I have seen consciousness perfect and sensibility entirely gone, and *vice versa.* The rule then should be, go always beyond the stage of excitement; till this is passed nothing can be done; but as soon as it is passed, pause, and arrest the process always short of stertor.

Such I believe to be the true practice in obstetrics, and under it *no fatal case has occurred.*

Now, may not the surgeons profit by our good and their evil fortune, and stop short of the deep, dangerous state of anæsthesia, into which they now plunge their patients? Something ought, nay, something *must* be done to prevent these ever recurring deaths from anæsthesia; and it does seem to me that those who can guide and control surgical opinion, ought to be willing to sacrifice the advantages of complete insensibility, that others may not jeopardize life. It is very true that one who has very large experience may again and again crowd patients down into deep snoring anæsthesia, and yet no harm come of it; but if he do it, the man of less knowledge and of narrower experience will do the same thing, and death to the patient and disgrace to the practitioner be the disastrous result.

There is another idea on this subject which presses upon my mind when I read these terrible cases, so strongly that I must give it utterance; and I hope that the thoughtful members of the profession, who feel as we all ought to feel, that nothing should be neglected that may by possibility free the skirts of the profession from the deep disgrace of these repeated deaths by chloroform, will not reject my proposition till they have well considered it. It is my deliberate opinion that, in every case of death from chloroform, the whole history of the case ought to be fully and impartially investigated by a coroner's jury. I know and feel the objections to this course. I know that it may—nay that it must fall occasionally with crushing force upon a brother practitioner, who may be entirely innocent of negligence or rashness—still I say it ought to be done. Let it fall where it may, the thing ought to be done. It will be a terror to evil doers, it will restrain the rash, it will punish the guilty; and that there has been fearful rashness and deep guilt in some of these cases, no man who has used chloroform often can doubt. If in every case the inexperienced would use ether; if, when chloroform was ventured on, a competent person had his fingers on the pulse, and his undivided attention fixed on the anæsthetic state of the patient, who believes that the fatal cases would have occurred? *I do not!* If, then, these precautions are neglected, is not such neglect criminal? I believe it is, and that the profession owe it to themselves to have the question of guilt or innocence impartially investigated. The time will soon come, when, if we do nothing, the public will demand this in every case. It is better that the profession propose, than that they be hereafter forced to submit to such investigations.—*New York Medical Times.*

